# Diversity of arbuscular mycorrhizal fungi and its response to seasonal variation in alpine grassland of the eastern Tibetan Plateau

**DOI:** 10.3389/fmicb.2025.1511979

**Published:** 2025-02-25

**Authors:** Wanqing Dong, Tingting Ding, Tingyu Duan

**Affiliations:** ^1^State Key Laboratory of Herbage Improvement and Grassland Agro-ecosystems, Lanzhou University, Lanzhou, China; ^2^Key Laboratory of Grassland Livestock Industry Innovation, Ministry of Agriculture and Rural Affairs, Engineering Research Center of Grassland Industry, Ministry of Education, Gansu Tech Innovation Center of Western China Grassland Industry, Lanzhou, China; ^3^College of Pastoral Agriculture Science and Technology, Lanzhou University, Lanzhou, China

**Keywords:** AM fungi, seasonal changes, Illumina HiSeq sequencing, soil factors, community composition

## Abstract

**Introduction:**

Arbuscular mycorrhizal (AM) fungi play a crucial role in maintaining diversity and ensuring the proper functioning of grassland ecosystems. A comprehensive understanding of the diversity, distribution patterns, and drivers of AM fungi in different habitats is essential for exploring the ecological roles in grassland ecosystems.

**Methods:**

In this study, we utilized high-throughput sequencing technology to explore the diversity of AM fungi and their distribution at an altitude of approximately 3,500 m in the alpine grassland of the eastern Tibetan Plateau. Additionally, we investigated the impacts of seasonal variation on AM fungal communities.

**Results:**

A total of 97 species of AM fungi, comprising 937 operational taxonomic units (OTUs) belong to 9 families and 10 genera, were identified from the soil samples. Notably, the genera *Glomus* and *Paraglomus* were the most abundant and dominant within the identified communities. The composition, distribution, and diversity of the AM fungal communities in the alpine grassland of the eastern Tibetan Plateau were significantly affected by seasonal variation (*p* < 0.05), with geographic distance being a determining factor. Total nitrogen (TN), soil organic matter (SOM), and pH were identified as the key soil factors driving changes in AM fungal communities.

**Discussion:**

The results demonstrated that the characteristics of AM fungal communities in the alpine grasslands of the eastern Tibetan Plateau were affected by seasonal variations and geographic location, and these findings are significant for the application of AM fungi in the restoration of grassland in similar ecosystems.

## 1 Introduction

Grasslands are vital ecosystems on Earth, providing a diverse range of ecological, economic, and social functions (Zhao et al., 2020). They are significant for environmental protection, biodiversity conservation, livestock economy development, and addressing global climate change (Bengtsson et al., 2019). Arbuscular mycorrhizal (AM) fungi, an important type of soil fungi, establish symbiotic relationships with the roots of numerous grassland plants (Miller et al., 2012). This symbiotic relationship not only enhances nutrient absorption for plants but also improves soil health, thereby maintaining the stability and development of ecosystems (Miller et al., 2012). AM fungi expand the contact area between plant roots and soil through the extension of hyphae. This expansion increases the absorption and transformation of essential nutrients, such as nitrogen and phosphorus, as well as water, ultimately promoting plant growth and resilience (Allen, 2007). Additionally, AM fungi can enhance the physical structure of the soil by secreting substances like glomalin, which promotes the formation of soil aggregates, improving soil moisture retention and aeration (Miller and Jastrow, 2000).

AM fungi can also significantly influence vegetation communities and ecosystems in grasslands (Zhang et al., 2012), and accelerate the establishment of plant communities in degraded grasslands (Torrez et al., 2016). During the process of grassland succession, AM fungi increase the richness and diversity of plants, particularly among late-successional species (Koziol and Bever, 2017). Moreover, AM fungi suppress the occurrence of poisonous plants by regulating the interspecific competition among grassland plants (Jin et al., 2011; Neuenkamp et al., 2019), and alleviate the negative impacts of environmental issues such as nitrogen deposition, climate change, and soil pollution (Lenoir et al., 2016; Kang et al., 2019; Cui et al., 2021). Additionally, in the restoration of degraded grasslands, the recovery of AM fungal communities is faster than plant communities, facilitating the early establishment of mycorrhizal plants and thus accelerating the grassland recovery process (Mao et al., 2019). Therefore, conducting in-depth research and providing protection for these valuable fungal resources is essential for enhancing the stability and productivity of grassland ecosystems.

The composition and diversity of microbial communities undergo significant changes with variations in environmental conditions, serving as “indicators” in ecosystems (Bissett et al., 2013). Among these communities, AM fungi are highly sensitive to environmental changes, and their community characteristics reflect the health status of grassland ecosystems. For example, the dominant species and distribution of AM fungi effectively indicate soil health and the extent of grassland degradation (Vasconcellos et al., 2016; Wu et al., 2021). This sensitivity has increased the value of AM fungi in environmental monitoring and ecological restoration efforts (Asmelash et al., 2016). The composition, diversity, and functional characteristics of AM fungal communities are influenced by various factors, including vegetation communities, soil physicochemical properties, climate change, and human activities (Mohammad et al., 2003; Jamiołkowska et al., 2018; Jerbi et al., 2021). Among these factors, the type of host plant is a key biological determinant affecting AM fungal communities (López-García et al., 2017). Additionally, other factors, such as soil pH, directly influence the growth, reproduction, and interactions of AM fungi with plants (Van Aarle et al., 2002; Li et al., 2021). Moreover, variations in soil nutrient content, particularly nitrogen and phosphorus, due to management practices or climate change significantly impact the structure and function of AM fungal communities (Treseder and Allen, 2002). Research indicates that soil available nitrogen can directly or indirectly alter the composition of AM fungi (Xiao et al., 2021; Shi et al., 2022), while soil available phosphorus shows a significant negative correlation with the richness of AM fungi (Ceulemans et al., 2019).

Recognizing the interrelated impacts of various environmental and anthropogenic factors on AM fungal communities is crucial, as these factors are often interconnected rather than isolated. For example, significant differences in the composition and diversity of AM fungal communities have been observed across altitudinal gradients. As altitude increases, changes in climate and soil conditions typically occur, directly impacting the distribution and activity of AM fungi. Additionally, these changes can produce indirect effects through alterations in vegetation communities (Gai et al., 2012; Geml, 2017). External conditions such as mowing, grazing, fertilization, and climate change have complex and patterned influences on AM fungal communities (Chen et al., 2014; Faghihinia et al., 2022; Zubek et al., 2022). Therefore, ongoing in-depth research on the responses of AM fungal communities to environmental changes will enhance our understanding of the adaptive mechanisms and future trends of grassland ecosystems.

The Tibetan Plateau, recognized as the highest geomorphological unit on Earth, is characterized by its unique geography, alpine climate, and natural geographic distribution pattern, which play a vital role in maintaining ecological stability (Dong et al., 2022). Alpine grasslands encompass over three-fifths of the plateau and mainly include alpine meadow and alpine steppe, which are the two most representative types of alpine grassland ecosystems (Duan et al., 2021). Over the past decades, the Tibetan Plateau has experienced the impacts of climate change (Latif et al., 2019), rodent foraging (Zhang et al., 2022) and overgrazing (Niu et al., 2019). These factors have led to a rapid decline in the diversity, structure, and function of grassland vegetation communities (Zhang et al., 2019). Additionally, there has been a loss of soil nutrients and degradation of soil structure, which hinders plant growth and reduces above-ground productivity (Zhang et al., 2019). The highly fragile nature of the alpine grassland ecosystem on the Tibetan Plateau makes restoration difficult once degradation has occurred (Dong, 2023). Therefore, research on restoration of degraded grasslands has gradually become a research hotspot.

At present, most studies on the diversity of AM fungi have been conducted in regions such as Tibet and Qinghai in the southwest and northwest. In contrast, relatively few investigations have been conducted in eastern areas such as Sichuan and Gansu (Zhang et al., 2017). Given the vast extent of the Tibetan Plateau and the significant habitat differences between different regions, further research is needed to expand the scope of research. Moreover, a multitude of studies have revealed that seasonal variation can affect spore density, root colonization, community composition, and diversity of AM fungi (Li et al., 2005; Lara-Pérez et al., 2020; Liu et al., 2020). However, little attention has been given to how seasonal variation affects the diversity and composition of AM fungal communities specifically on the Tibetan Plateau. In light of this gap, the current study used high-throughput sequencing technology to explore the composition of AM fungal communities and their seasonal dynamics in alpine grasslands of the eastern Tibetan Plateau. Additionally, the relationships between soil properties, seasonal variation, and AM fungal communities were analyzed. We hypothesized that seasonal variations modulate soil properties, thereby indirectly affecting the structure and composition of AM fungal communities.

## 2 Materials and methods

### 2.1 Study region

The research sites are located in the Aba Tibetan and Qiang Autonomous Prefecture, situated on the southeastern edge of the Tibetan Plateau, and constitutes one of the principal distribution regions of natural grasslands in Sichuan Province. The main forms of grassland degradation at the sites include desertification and the emergence of black soil patches. The present study selected 2 distinct types of degraded grasslands in Aba Prefecture in the eastern Tibetan Plateau. Site 1 is located in Hongyuan county (HY, 32°53′7″ - 32°53′25″ N, 102°35′58″ - 102°36′2″ E) and is mainly afflicted by desertification. The dominant plants are *Kobresia setchwanensis* and *K. pygmaea*, with accompanying species such as *Potentilla anserina*, *Aster alpina*, *Anemone trullifolia*, *A. rivularis*, and *Thalictrum alpinum*. Site 2 is located in Ruoergai county (R, 33°55′40″-33°55′43″ N, 102°23′30″-102°23′33″ E) and is mainly characterized by bared black soil patches. The dominant plants are *Carex moorcroftii* and *K. pygrwaea*, with subsidiary species such as *Elymus nutans, Leymus paboanus* and *Artemisia wellbyi*. Both sites exhibit a moderate degree of degradation and are situated at an elevation of approximately 3,500 m. The mean annual temperature ranges from 1.1 to 2.9°C, while the average annual precipitation varies from 648 to 860 mm, indicative of a continental plateau cold temperate monsoon climate.

### 2.2 Sample collection

To evaluate the AM fungal community and its seasonal response in the study area, soil samples were collected in July (Summer, S) and September (Autumn, A) in the year 2020 and 2021 from two sites: Hongyuan (HY) and Ruoergai (RA) in the alpine grassland on the Tibetan Plateau. The eight sampling events are designated as follows: HYS20 for Hongyuan Summer 2020, RS20 for Ruoergai Summer 2020, HYA20 for Hongyuan Autumn 2020, RA20 for Ruoergai Autumn 2020, HYS21 for Hongyuan Summer 2021, RS21 for Ruoergai Summer 2021, HYA21 for Hongyuan Autumn 2021, and RA21 for Ruoergai Autumn 2021. Within each site, 12 sampling points were randomly selected, each at least 100 m apart, with each sampling point considered a replicate. At each sampling point, 3 soil samples (0–15 cm) were randomly collected using a shovel and thoroughly mixed to form a composite sample. A total of 96 soil samples were collected (2 sites ×2 seasons ×2 years ×12 replicates). Fresh soil samples had visible plant residues, roots, and stones removed and were then passed through a sieve (< 2 mm). The sieved soil samples were split into two parts. One subsample was used for sequencing, while the other was air-dried to evaluate the basic properties of the soil.

### 2.3 Soil property measurements

Refer to Lu (2000) for the determination of soil properties. Soil pH was measured using a pH meter (1∶2.5, V_soil_ ∶ V_water_) (PHS-3C, Shanghai Yidian, China). Total nitrogen (TN) content in soil was determined through acid digestion and the Kjeldahl method using a decoction oven (SH220F, Shandong Haineng, China) and an Automated Discrete Analyzer (SMARTCHEM 450, Beijing Lijia, China). Total phosphorus (TP) content in soil was dissolved in a mixture of concentrated H_2_SO_4_ and HClO_4_ using a decoction oven (SH220F, Shandong Haineng, China) and assessed according to the molybdate-blue colorimetric method in an Automated Discrete Analyzer (SMARTCHEM 450, Beijing Lijia, China). Available potassium (AK) content in soil was extracted with NH_4_OAc and measured using a Flame Photometer (Model 410 Flame Photometer, Sherwood Scientific, UK). Soil organic matter (SOM) content was determined by the potassium dichromate oxidation method using an oil bath (HH-S, Changzhou Putian, China).

### 2.4 Illumina HiSeq sequencing and bioinformatics analyses

Illumina HiSeq sequencing was employed to investigate the community diversity and distribution of AM fungi. Firstly, soil DNA was extracted from 0.5 g of fresh soil using the E.Z.N.A.^®^ soil DNA kit (Omega Biotek, United States) following the manufacturer’s instructions. Purity and concentration were assessed using a NanoDrop 2000 ultra-micro spectrophotometer (Thermo Scientific, Inc., United States). The V4-V5 hypervariable regions of the fungal gene (18S rRNA) were amplified with primers AML1 and AML2 in a thermal cycle PCR system, followed by a second amplification using the primers AMV4.5NF and AMDGR (Lee et al., 2008; Suzuki et al., 2020). In the PCRs, a mixture consisting (20 μL) of 5 × FastPfu Buffer (4 μL), 2.5 mM dNTPs (2 μL), 0.5 μM each primer (0.8 μL), FastPfu Polymerase (0.4 μL), BSA (0.2 μL) and Template DNA (10 ng), was utilized for the reactions. The standard procedure for database building operations was conducted according to the NEBNext^®^ Ultra^™^ DNA Library Prep Kit for Illumina^®^ (New England Biolabs, United States). Amplicon sequencing was performed using Illumina Hiseq2500 platform (Meige Company, Shenzhen, China).

Finally, the sequencing data were processed. The quality of the Raw Reads data was filtered at both ends independently using the Trimmomatic software (V0.33). Leveraging the primer and barcode information at the start and end of the sequences, the sequences were allocated to the corresponding samples with the help of Mothur software (V1.35.1). Subsequently, the barcodes and primers were removed, thereby obtaining paired-end clean reads after quality control. Next, the PE reads were spliced by means of FLASH (V1.2.11) software and filtered to acquire the raw spliced sequences (raw tags). The spliced sequences were subject to quality control and filtration using Mothur software to obtain valid spliced fragments (Clean Tags). OTUs clustering was then performed and 18S reads were purified from the MaarjAM database (Opik et al., 2010). Subsequently, the OTU was assigned to different fungal taxa (BLAST), with a 97% percentage similarity (Altschul et al., 1990). The sequence data has been uploaded to the NCBI database (BioProject ID: PRJNA1173197).

### 2.5 Statistical analyses

Based on the Richness-rarefaction curve, the sequencing depth in this study is sufficient (Supplementary Figure S1). We compared the number of OTUs and community composition at different sites and their seasonal dynamics using the original OTU data. Subsequently, based on the minimum sequence, we rarefied the original OTU table, and the resulting data were utilized to analyze the Alpha diversity, Beta diversity, differential microbial communities, and correlation analysis of AM fungi. R software (V2.15.3) was utilized to conduct statistics on the relative abundances of OTUs, followed by the creation of a bar chart depicting community composition. Subsequently, QIIME software (V1.9.1) was employed to calculate four diversity indices (Chao1, Richness, Shannon, Simpson). To evaluate effects of season, site, their interactions, and year on the composition of the AM fungal community, we utilized “strata” in the “adonis” function to account for difference in site and season. Furthermore, to illustrate the difference in AM fungal community composition for site × season, Principal Coordinate Analysis (PCoA) was employed to order the Bray–Curtis dissimilarity matrix.

For the difference analysis of species between samples, we used LEfSe. First, the taxa with significant abundance difference between samples were compared and screened. Next, the paired Wilcoxon rank-sum test was employed to assess the differences between each pair of samples. Finally, Linear Discriminant Analysis (LDA) was utilized for dimensionality reduction and to evaluate the impact magnitude of taxa with significant differences (LDA > 2), followed by plotting. Redundancy Analysis (RDA) was conducted to explore the relationship between AM fungal community characteristics and soil properties. Pearson’s correlations analysis was employed to investigate the relationships between AM fungi and soil properties. The Co-occurrence network diagrams were calculated and plotted using R (version 4.2.3) and Gephi (version 0.9.2) (threshold = 0.0001). Bar graphs were plotted in GraphPad Prism 8.0, while Venn diagrams, PCoA, RDA, and Heatmaps were generated using Wekemo Bioincloud^1^ (Gao et al., 2024). One-way Analysis of Variance (ANOVA) was used to assess soil properties and AM fungal diversity (SPSS, version 22.0). At the 5% significance level, the Tukey (HSD) test was employed for comparative analysis of the significance of different samples.

## 3 Results

### 3.1 Soil properties

There were significant differences in soil properties between sites and seasons as measured over 2 years (Table 1). In 2020, the contents of TN, TP and SOM in the soil ranged from 5.01–14.10, 0.82–1.00, and 8.56–18.09 g/kg, respectively, while the contents of AK in the soil ranged from 81.35–121.55 mg/kg. Seasons (summer vs. autumn) did not affect TP or AK, however, TN and SOM exhibited significant differences. The TN and SOM in summer soils were significantly higher those in autumn soils (*p* < 0.05). Moreover, site location (Hongyuan vs. Ruoergai) had no effect on TP, but TN, SOM and AK showed significant differences. The TN in Hongyuan was significantly lower than in Ruoergai in summer soils (*p* < 0.05), while no significant difference was observed in autumn soils. Additionally, SOM and AK in Hongyuan were significantly lower than those in Ruoergai across all soil samples (*p* < 0.05). The soil pH ranged from 4.48 to 5.41 in Hongyuan and from 6.48 to 7.49 in Ruoergai. Consistent with TN, the pH of summer soil samples was significantly higher than that of autumn soil samples (*p* < 0.05).

**Table 1:** AM fungi alpha-diversity indices in all soil samples.

		Chao1	Richness	Shannon	Simpson
2020	HYS20	142.33 ± 10.84b	139.83 ± 10.8b	1.02 ± 0.07b	0.24 ± 0.04a
	HYA20	144.94 ± 3.96b	141.17 ± 4.28b	1.14 ± 0.02b	0.15 ± 0.01b
	RS20	167.54 ± 7.5ab	164.17 ± 7.53ab	0.87 ± 0.04c	0.29 ± 0.03a
	RA20	192.47 ± 10.55a	189.5 ± 10.58a	1.29 ± 0.04a	0.09 ± 0.01b
2021	HYS21	76.65 ± 2.34c	73.33 ± 1.97c	0.86 ± 0.08b	0.33 ± 0.06a
	HYA21	115.42 ± 5.14b	110.25 ± 5.31b	0.91 ± 0.04b	0.23 ± 0.02ab
	RS21	72.3 ± 3.27c	70.42 ± 3.28c	1.23 ± 0.05a	0.14 ± 0.03b
	RA21	148.98 ± 7.87a	145.25 ± 8.33a	1.17 ± 0.03a	0.14 ± 0.01b

Data are presented as means ± SE (*n* = 12). Significant differences among treatments for each variable were assessed using Tukey’s honestly significant difference (HSD) test (*p* < 0.05) following one-way ANOVA and are indicated by different letters. HYS20, HYA20, RS20, RA20, HYS21, HYA21, RS21, and RA21 represent different groups of soil samples.

In 2021, the contents of TN, TP, and SOM in soil samples ranged from 2.54–6.21, 1.01–1.21, and 4.62–13.82 g/kg, respectively, while AK contents ranged from 69.49–111.53 mg/kg. In Hongyuan, the season significantly affected TN, TP, and SOM content, however AK showed no significant differences. In Ruoergai, the season had no effect on TN, TP, SOM, and AK. Additionally, TN and SOM were significantly higher in Hongyuan than in Ruoergai for both summer and autumn soils (*p* < 0.05). For TP and AK, levels were significantly higher in HYS than in RS (*p* < 0.05), with no significant difference between HYA and RA. The soil pH ranged from 5.71–6.04 in Hongyuan and from 7.42–7.61 in Ruoergai. In Hongyuan, the pH of summer samples was significantly higher than that of autumn samples (*p* < 0.05), while no significant difference was observed in Ruoergai. These results showed that seasonal variation significantly affected the physicochemical properties of the soils in Hongyuan and Ruoergai, particularly TN, TP, SOM and pH.

### 3.2 AM fungal community composition and structure

We characterized 15,933 OTUs (operational taxonomic units) of AM fungi using a 97% sequence similarity cutoff from 449,063 to 66,657 high-quality reads of the 96 soil samples. Most OTUs of AM fungi were classified as *Glomus* (15.73%), *Paraglomus* (15.60%), *Claroideoglomus* (5.06%), *Diversispora* (2.49%), followed by *Entrophospora* (2.15%), *Ambispora* (2.71%), and Others (1.52%) at the genus level (Supplementary Table S1). A total of 937 OTUs were detected, belonging to 4 order, 9 families, 10 genera and 97 species (Supplementary Table S2). Of the 97 species, 50 belonged to the genus *Glomus*, accounting for 51.55% of the total, making it the dominant genus. The remaining genera, in order from highest to lowest, are *Acaulospora*, *Claroideoglomus*, *Archaeospora*, *Diversispora*, *Scutellospora*, *Paraglomus*, *Ambispora*, *Entrophospora*, and *Pacispora*, which account for 11.34, 11.34, 5.15, 5.15, 5.15, 5.15, 3.09, 1.03, and 1.03% of the total, respectively (Supplementary Figure S2).

**Figure 1:**
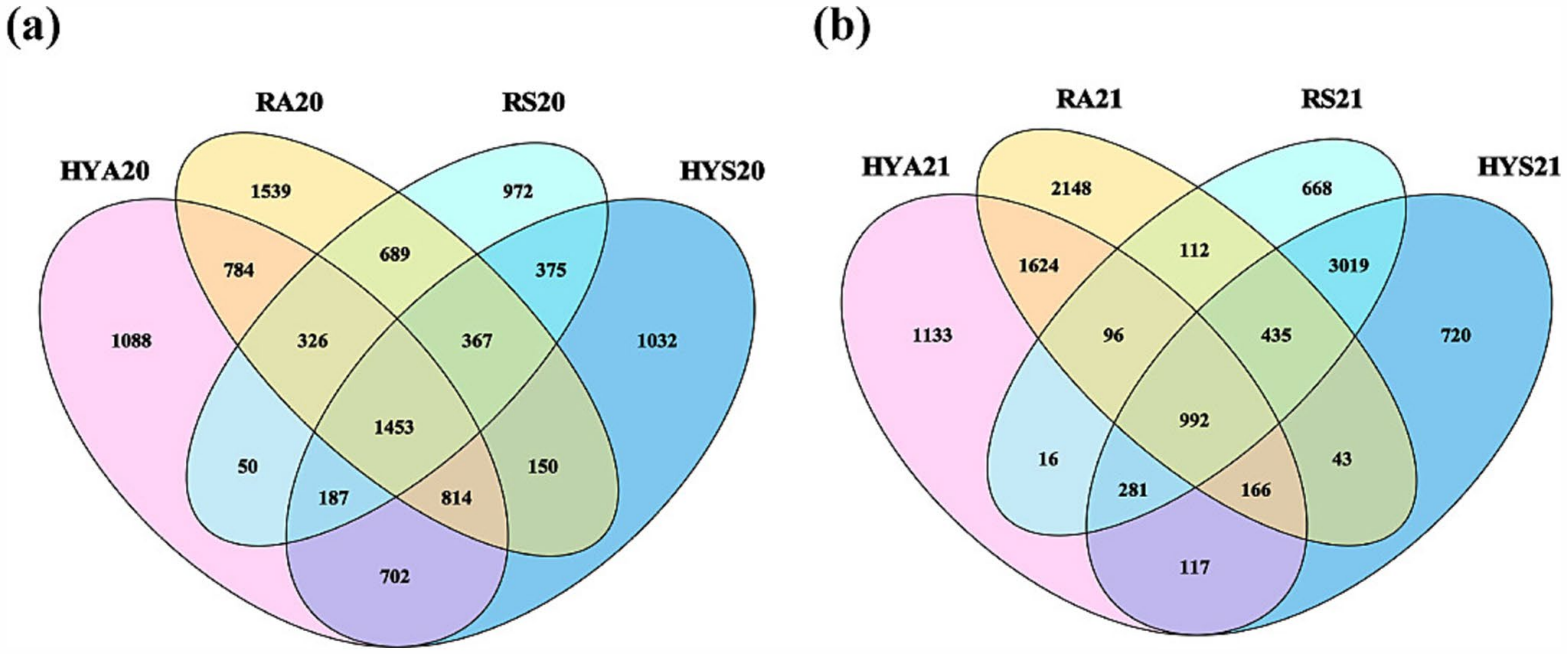
Venn. Number of unique and shared OTUs across different sites and seasons in 2020 **(A)** and 2021 **(B)**. HYS20, HYA20, RS20, RA20, HYS21, HYA21, RS21, and RA21 represent different groups of soil samples.

The number of unique and shared OTUs varied across different seasons and sites in 2020 and 2021 (Figure 1). In 2020, we identified 1,453 shared OTUs (Figure 1A), while in 2021, this number decreased to 992 (Figure 1B). The trend for unique OTUs was consistent in both years: RA > HYA > HYS > RS. Subsequently, we analyzed the number of AM fungal species in different soil samples (Supplementary Figure S3). The number of AM fungal species showed a gradual increase over time in Hongyuan, whereas no similar variation was observed in Ruoergai. In addition, in 2020, the number of AM fungal species in autumn soils from Hongyuan was significantly higher than in summer samples (HYA20 > HYS20) (*p* < 0.05), but no significant differences were found in 2021. In Ruoergai, there was no significant variation in the number of AM fungal species across different years and seasons. Thus, the effect of seasonal variation on the soil AM fungal community appears to differ among sites.

**Figure 2:**
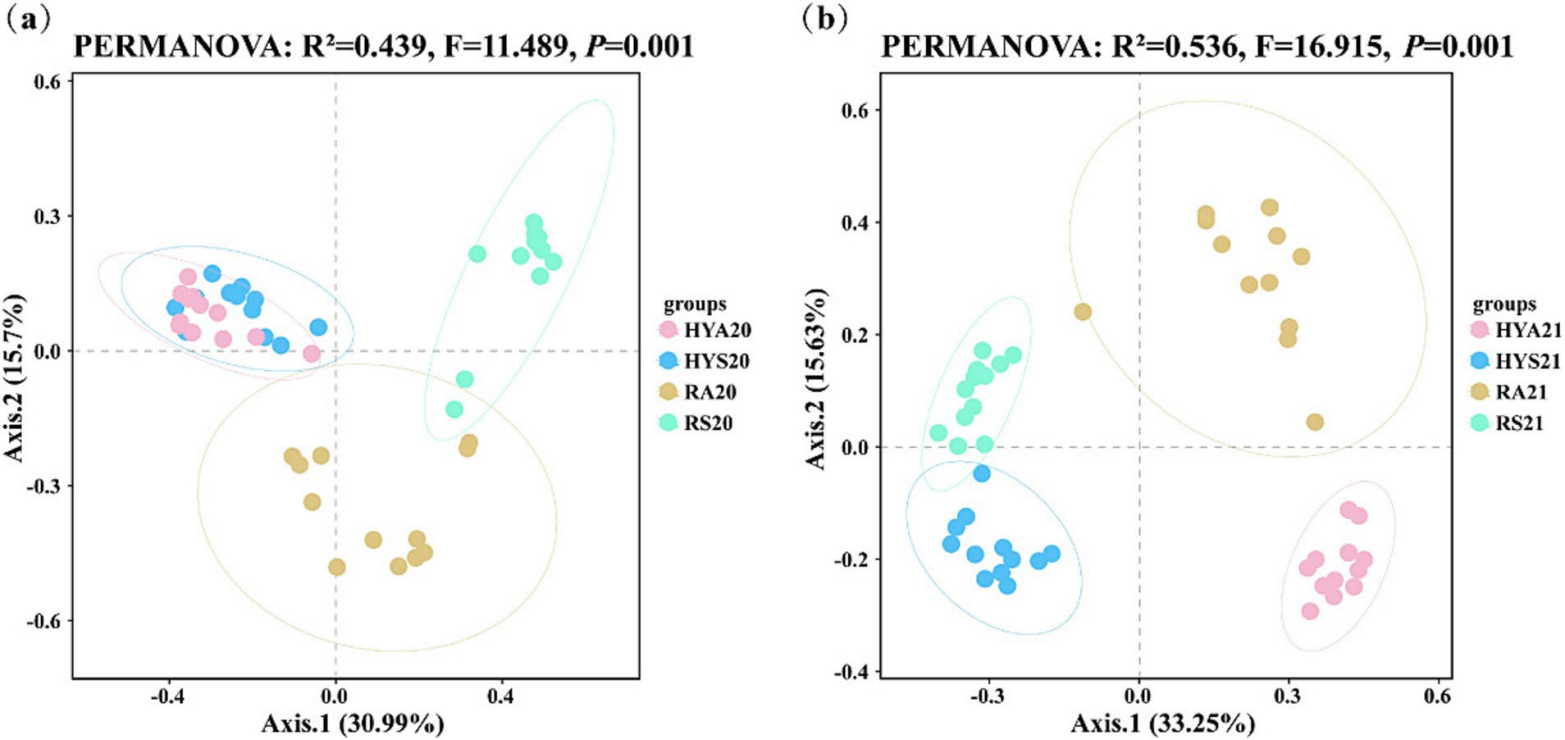
The principal coordinates analysis (PCoA) of AM fungal communities from soil samples collected in 2020 **(A)** and 2021 **(B)** is based on the Bray-Curtis distances. HYS20, HYA20, RS20, RA20, HYS21, HYA21, RS21, and RA21 represent different groups of soil samples.

To illustrate difference in the AM fungal community for site × season, we applied a PCoA using the Bray-Curtis algorithm (Figure 2). The PCoA assigned the AM fungal community to two groups: (HYS and HYA) and (RS and RA) in 2020 (Figure 2A). The HYS20 and HYA20 were clustered together and were distant from RS20 and RA20. In 2021, the PCoA assigned the AM fungal community to four groups: HYS, HYA, RS and RA (Figure 2B), which were distant from one another. Thus, the AM fungal community structure significantly changed with the season in 2020 and significantly changed with both season and site in 2021. PERMANOVA revealed that the interaction of season and site significantly affected the AM fungal community in both 2020 and 2021 (*p* = 0.001).

**Figure 3:**
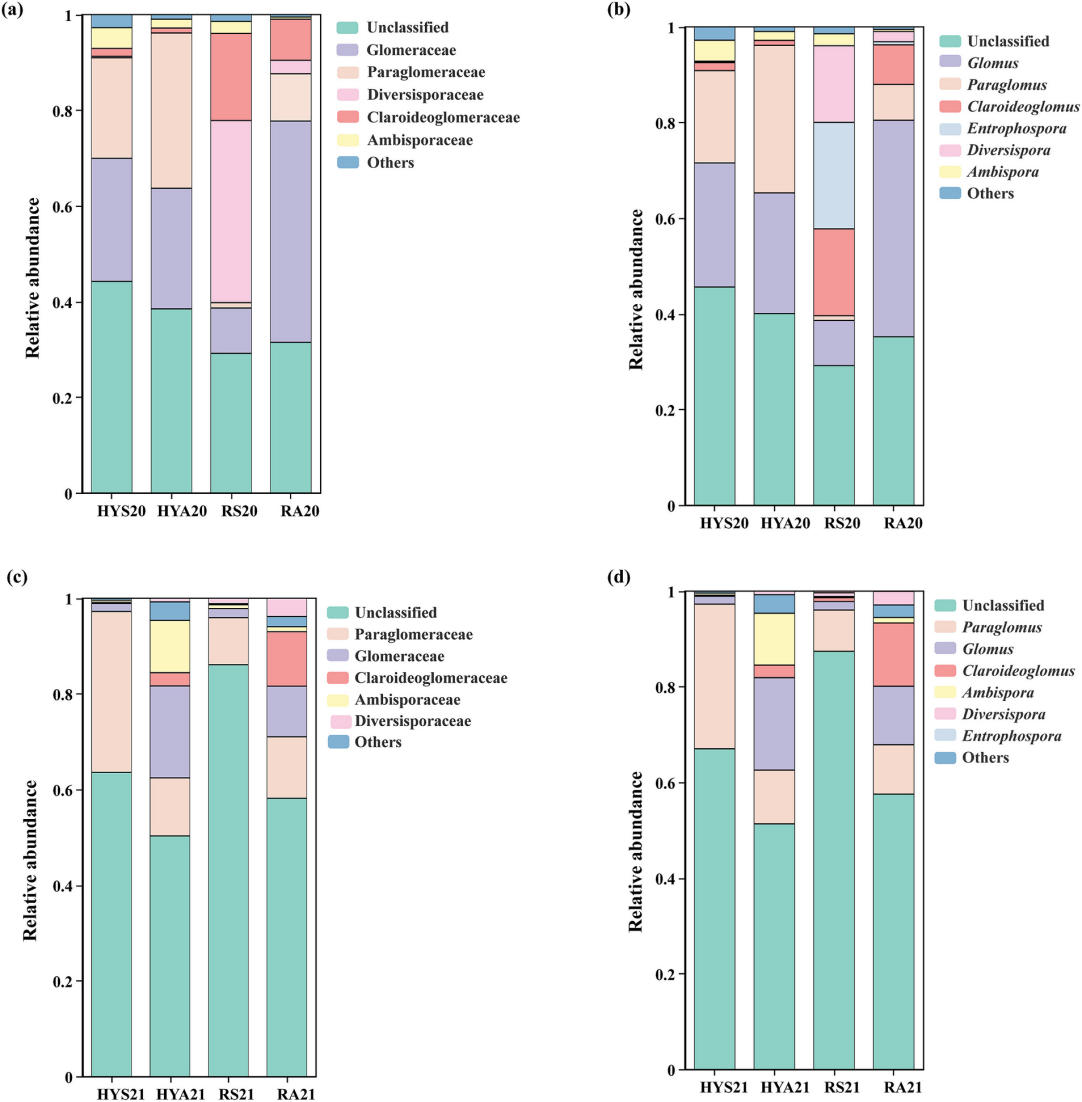
Community composition of AM fungi. The relative abundance (%) of AM fungi (based on the number of OTUs) at the family and genus levels in 2020 **(A, B)** and 2021 **(C, D)**. HYS20, HYA20, RS20, RA20, HYS21, HYA21, RS21, and RA21 represent different groups of soil samples. Others, Taxa with an abundance of <1% are summarized as “others,” including Archaeosporaceae (*Archaeospora*), Acaulosporaceae (*Acaulospora*), Gigasporaceae (*Scutellospora*), and Pacisporaceae (*Pacispora*).

Additionally, we analyzed community composition at the family and genus levels based on the relative abundance of AM fungi in soil samples (Figure 3). In 2020, the relative abundance of Paraglomeraceae (*Paraglomus*) was higher, whereas that of Ambisporaceae (*Ambispora*) and Claroideoglomeraceae (*Claroideoglomus*) were lower in HYA20 compared to HYS20. The relative abundance of Glomeraceae (*Glomus*) and Paraglomeraceae (*Paraglomus*) were higher, while that of Ambisporaceae (*Ambispora*), Claroideoglomeraceae (*Claroideoglomus*), and Diversisporaceae (*Diversispora*) were lower in RA20 than in RS20. In 2021, the relative abundance of Ambisporaceae (*Ambispora*), Claroideoglomeraceae (*Claroideoglomus*), and Glomeraceae (*Glomus*) was higher, whereas that of Paraglomeraceae (*Paraglomus*) were lower in HYA21 than HYS21. The relative abundance of Ambisporaceae (*Ambispora*), Claroideoglomeraceae (*Claroideoglomus*), Diversisporaceae (*Diversispora*), and Glomeraceae (*Glomus*) was higher in RA21 than in RS21. This result was contrary to that of 2020. These findings indicate that the community composition of AM fungi in the soils of Hongyuan and Ruoergai varies by years and is significantly influenced by seasonal variation.

**Figure 4:**
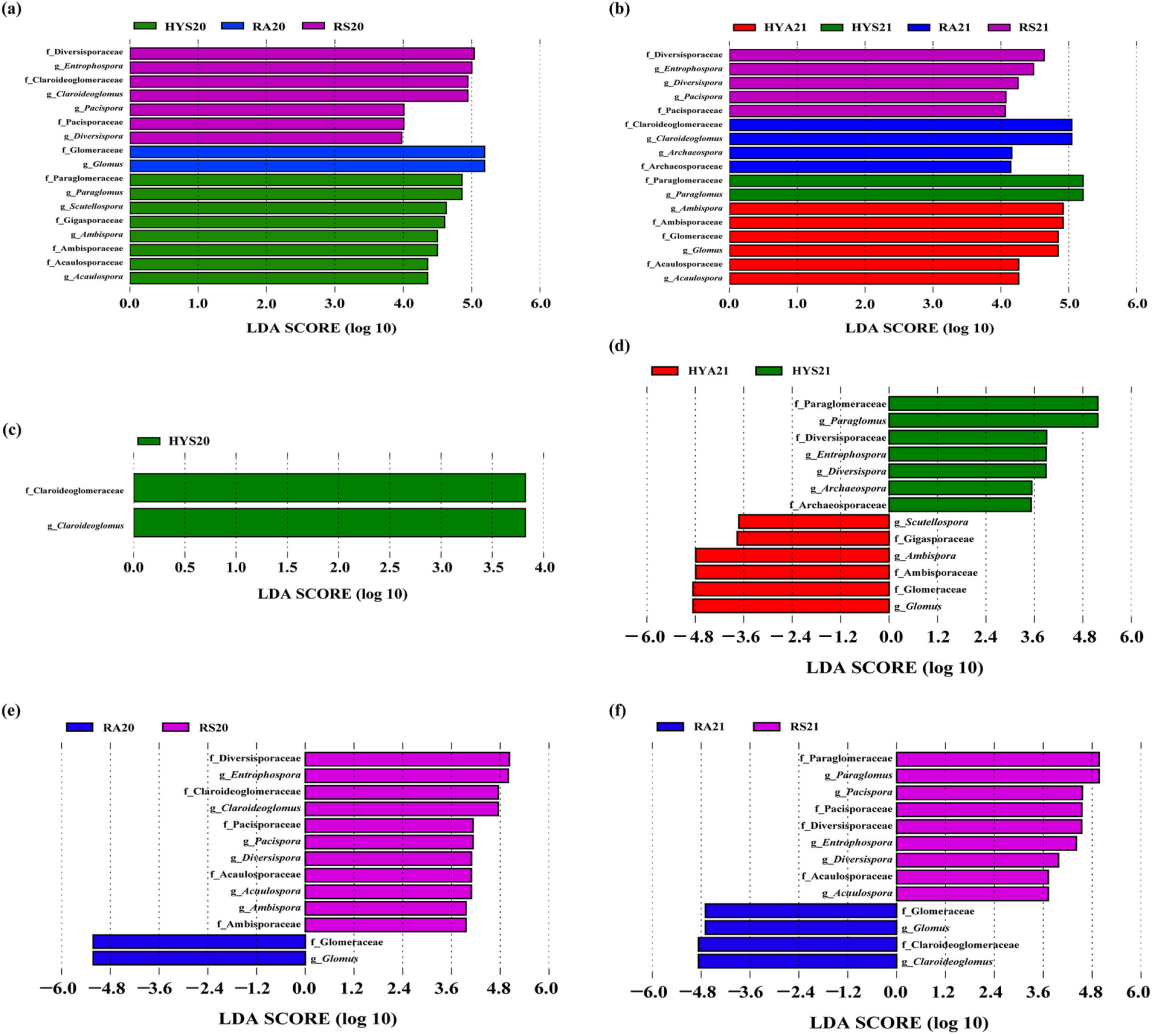
LEfSe analysis of AM fungal communities in 2020 **(A)** and 2021 **(B)** shows LDA scores exceeding 2.0 across all soil samples. **(C–F)** Represent the AM fungal taxa that exhibited significant changes due to seasonal variations across different years and sites. The LDA bars represent AM fungal taxa (at the family and genus levels) with LDA scores greater than 2.0 in soil samples from different sites and seasons (*p* < 0.05). HYS20, HYA20, RS20, RA20, HYS21, HYA21, RS21, and RA21 represent different groups of soil samples.

To identify the key differential species of AM fungi in different soil samples, we conducted a LEfSe analysis (Figure 4; Supplementary Figure S4). The linear discriminant analysis (LDA) of LEfSe across all soil samples indicated that more AM fungal taxa (4 families and 4 genus) were detected in the HYS20 compared to the other three subgroups in 2020 (Figure 4A). Additionally, the summer soil samples exhibited a greater number of differential AM fungal taxa than those in autumn (HYS20 > HYA20; RS20 > RA20). In 2021, the LEfSe analysis revealed that more AM fungal taxa (3 families and 3 genus) were detected in the HYA21 than in the other three subgroups (Figure 4B). Furthermore, the autumn soil samples in Hongyuan contained more differential AM fungal taxa than the summer samples, while the reverse was true in Ruoergai. We further demonstrated that seasonal variation significantly affected the community composition of AM fungi in both Hongyuan and Ruoergai, particularly in the latter, with effects varying between years.

Subsequently, we analyzed the AM fungal taxa in Hongyuan and Ruoergai that exhibited significantly changes due to seasonal influences across different years (Figures 4C–F). In Hongyuan, there were 2 and 13 AM fungal taxa with significant changes in 2020 and 2021, respectively. Specifically, the AM fungal taxa that changed significantly under seasonal influence included 1 family and 1 genus: Claroideoglomeraceae (*Claroideoglomus*) in 2020 (Figure 4C). In 2021, the differential AM fungal taxa included 6 families and 7 genera, namely Archaeosporaceae (*Archaeospora*), Ambisporaceae (*Ambispora*), Diversisporaceae (*Diversispora*), *Entrophospora*, Gigasporaceae (*Scutellospora*), Glomeraceae (*Glomus*), and Paraglomeraceae (*Paraglomus*) (Figure 4D). In Ruoergai, there were 13 AM fungal taxa with significant changes in 2020 and 2021, respectively. The differential AM fungal taxa included 6 families and 7 genera, namely Acaulosporaceae (*Acaulospora*), Ambisporaceae (*Ambispora*), Claroideoglomeraceae (*Claroideoglomus*), Diversisporaceae (*Diversispora*), *Entrophospora*, Glomeraceae (*Glomus*), and Pacisporaceae (*Pacispora*) in 2020 (Figure 4E). The differential AM fungal taxa consisted of 6 families and 7 genera: Acaulosporaceae (*Acaulospora*), Claroideoglomeraceae (*Claroideoglomus*), Diversisporaceae (*Diversispora*), *Entrophospora*, Glomeraceae (*Glomus*), Paraglomeraceae (*Paraglomus*), and Pacisporaceae (*Pacispora*) in 2021 (Figure 4F). This result serves as an important data support for subsequent analysis and research.

### 3.3 AM fungal richness and diversity

We calculated the Chao1, Richness, Shannon and Simpson indices of AM fungi to evaluate the α-diversity of the AM fungal community in 2020 and 2021 across different sites and seasons (Table 2). The results showed that the Chao1, Richness, Shannon and Simpson indices of AM fungal communities in soil samples varied significantly between sites and seasons (*p* < 0.01), although the trends differed between years.

**Table 2:** Comparison of soil properties under different sites and seasons.

		TN (g/kg)	TP (g/kg)	SOM (g/kg)	AK (mg/kg)	pH
2020	HYS20	10.54 ± 1.15b	0.88 ± 0.05ab	13.79 ± 1.34b	81.35 ± 8.24b	5.41 ± 0.05c
	HYA20	5.01 ± 0.22c	1.00 ± 0.03a	8.56 ± 0.43c	87.01 ± 6.43b	4.48 ± 0.05d
	RS20	14.10 ± 0.52a	0.82 ± 0.06b	18.09 ± 0.50a	121.55 ± 10.07a	7.49 ± 0.03a
	RA20	7.60 ± 0.58c	0.84 ± 0.03ab	14.46 ± 1.15b	119.62 ± 20.31a	6.48 ± 0.20b
2021	HYS20	4.04 ± 0.10b	1.21 ± 0.02a	8.29 ± 0.20b	111.53 ± 9.89a	5.71 ± 0.05c
	HYA20	2.54 ± 0.18c	1.04 ± 0.03b	4.62 ± 0.31c	85.43 ± 7.14ab	6.04 ± 0.04b
	RS20	6.21 ± 0.30a	1.02 ± 0.02b	13.82 ± 0.69a	69.49 ± 2.88b	7.61 ± 0.02a
	RA20	6.09 ± 0.24a	1.01 ± 0.02b	12.28 ± 0.35a	83.94 ± 7.08b	7.42 ± 0.10a

Data are presented as means ± SE (*n* = 12). Significant differences among treatments for each variable were assessed using Tukey’s honestly significant difference (HSD) test (*p* < 0.05) following one-way ANOVA and are indicated by different letters. HYS20, HYA20, RS20, RA20, HYS21, HYA21, RS21, and RA21 represent different groups of soil samples. TN, total nitrogen; TP, total phosphorus; AK, available potassium; SOM, soil organic matter; pH, soil pH.

In 2020, the Chao1 and Richness indices of AM fungal communities in autumn soils were similar to those in summer soils. The Shannon index was higher in autumn soils compared to summer soils, with significant differences observed in Ruoergai (*p* < 0.05). Conversely, the Simpson index in autumn soil samples was significantly lower than in summer samples (*p* < 0.05). Additionally, the Chao1, Richness and Shannon were significantly higher in RA20 than in HYA20 (*p* < 0.05), while Shannon index was significantly lower in RS20 than in HYS20 (*p* < 0.05).

In 2021, the Chao1 and Richness indices of AM fungal communities in autumn soils were significantly higher than in summer soils (HYA21 > HYS21; RA21 > RS21) (*p* < 0.05). The Shannon index in autumn soils was similar to those in summer soils in Hongyuan. The Simpson index was higher in HYS21 than in HYA21. The Chao1, Richness, and Shannon were significantly higher in RA21 than in HYA21 (*p* < 0.05), Shannon was significantly higher in RS21 than in HYS21 (*p* < 0.05), and Simpson was significantly lower in RS21 than in HYS21 (*p* < 0.05). These findings indicate potential site-specific environmental factors influencing AM fungal communities. Furthermore, the significant differences in diversity indices within the same season across different sites suggest that local conditions, such as soil properties and microclimates, play a crucial role in shaping AM fungal diversity.

### 3.4 AM fungal network structure

**Figure 5:**
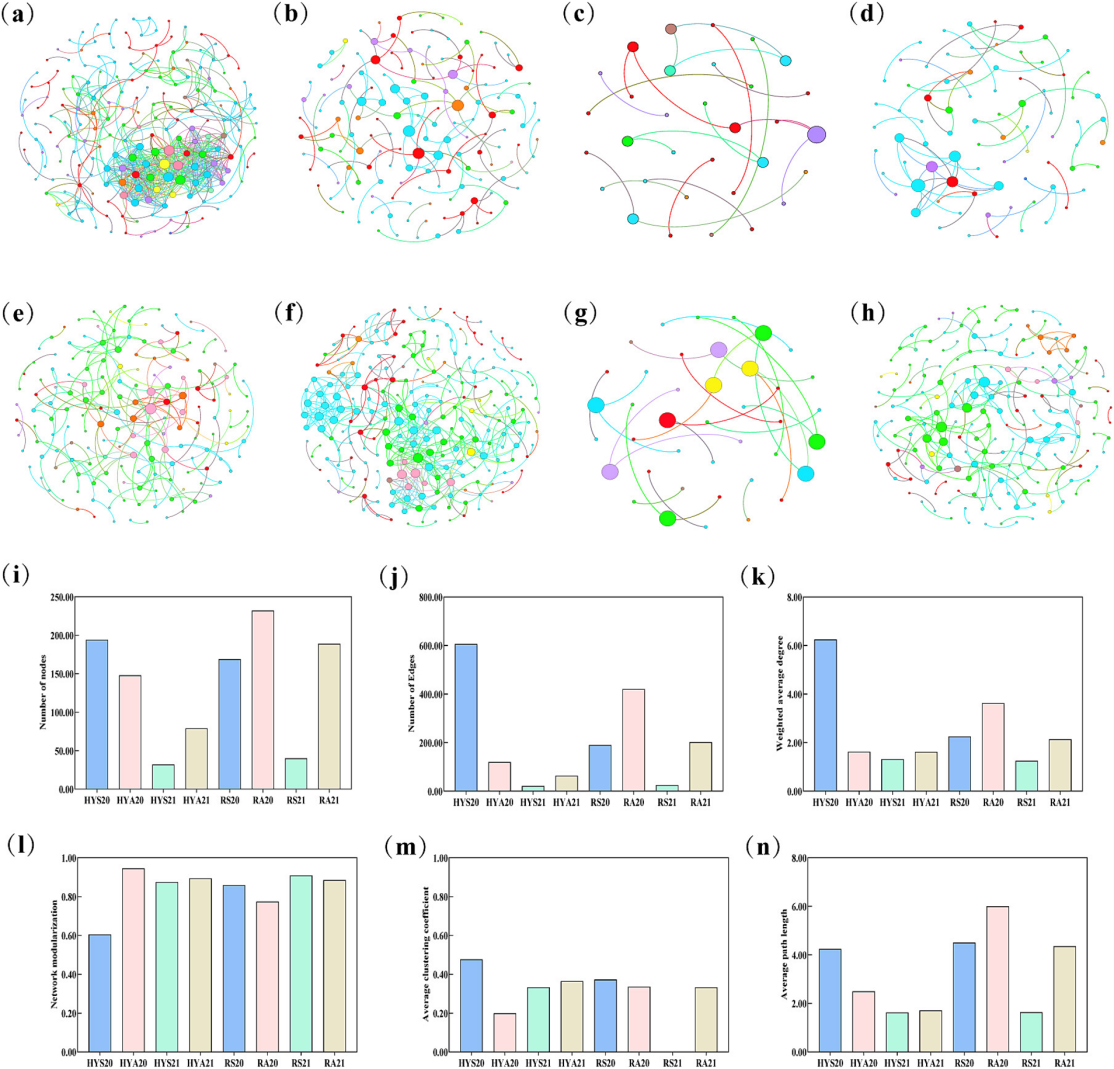
Network structure analysis of AM fungal community. **(A–H)** Represent the co-occurrence pattern of HYS20, HYA20, HYS21, HYA21, RS20, RA20, RS21, and RA21, respectively. Nodes are colored according to AM fungal taxa at the genus level, while the width and color of edges indicate the correlation coefficient. Different colors represent different AM fungal genera. **(I–N)** Represent different network topology properties, including the number of nodes, number of edges, weighted average degree, modularity, average clustering coefficient, and average path length.

The network structure based on the OTU data of AM fungal revealed differences between sites and seasons, with the AM fungal in Ruoergai exhibiting a significantly more complex network than those in Hongyuan (Figure 5). We further analyzed and compared the number of nodes, edges, weighted average degree, modularity, average clustering coefficient, and average path length across eight network structures (Figures 5I–N). The results indicated that the number of nodes, edges, weighted average degree, average clustering coefficient, and average path length were higher in HYS20 than in HYA20, while these properties were lower in HYS21 than in HYA21. The network modularization was lower in HYS20 than in HYA20, but higher in HYS21 than in HYA21. In Ruoergai, the number of nodes, edges, weighted average degree, and average path length were lower in summer soils than in autumn soils (RS20 < RA20; RS21 < RA21). Conversely, the network modularization was higher in summer soils than in autumn soils (RS20 > RA20; RS21 > RA21). The average clustering coefficient was higher in RS20 compared to RA20, while it was lower in RS21 than in RA21. These findings suggest that seasonal variation influences the complexity of AM fungal communities in the soils of Hongyuan and Ruoergai.

### 3.5 Correlations of AM fungal community with soil properties

**Figure 6:**
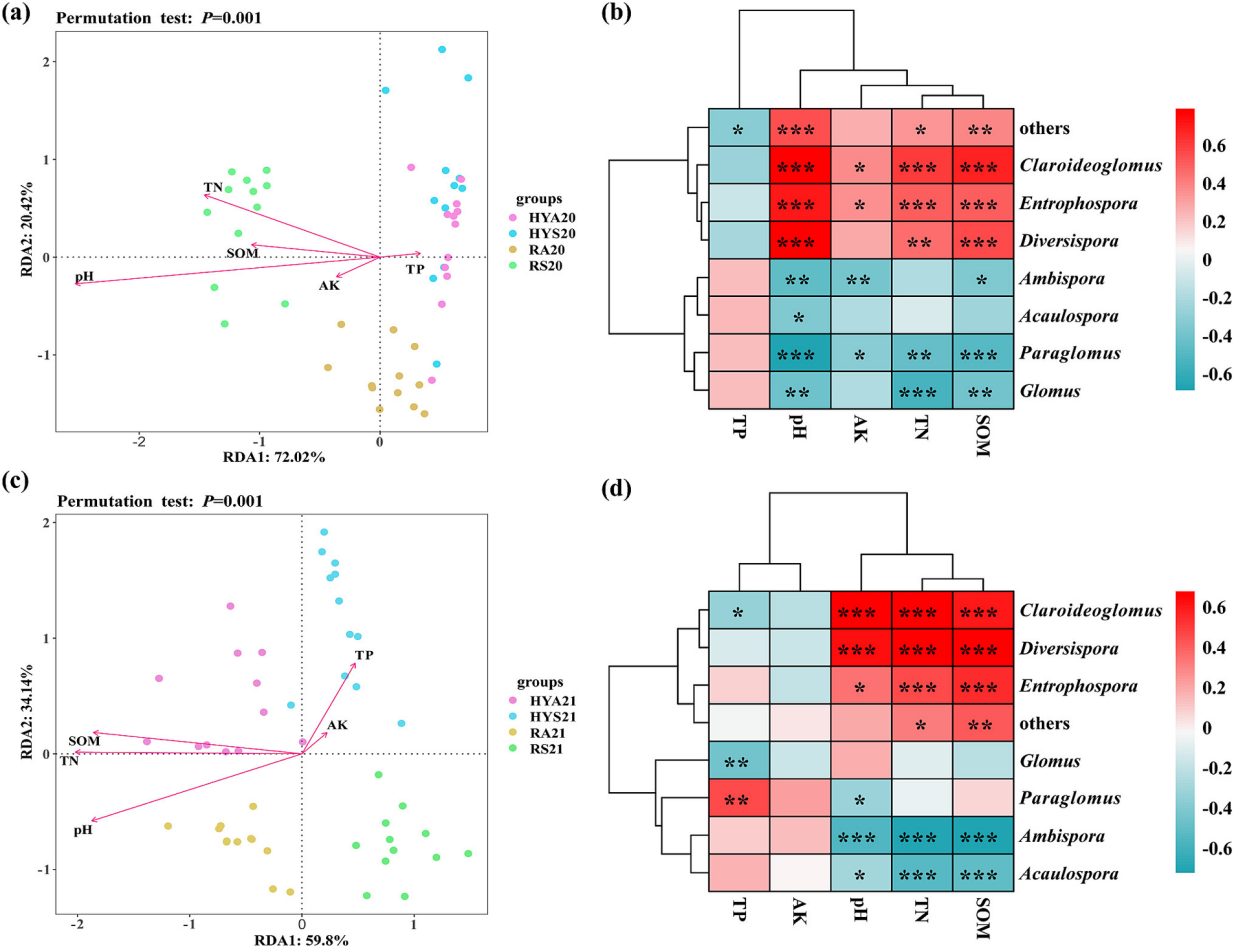
Redundancy analysis (RDA) at the genus level and Spearman’s rank correlation heatmap (*p* < 0.05 *, *p* < 0.01 **, *p* < 0.001 ***), were employed to examine the correlation between AM fungal communities and soil properties in 2020 **(A,B)** and 2021 **(C,D)**. The genera with abundance <1% are summarized as “others.” HYS20, HYA20, RS20, RA20, HYS21, HYA21, RS21, and RA21 represent different groups of soil samples. TN, total nitrogen; TP, total phosphorus; AK, available potassium; SOM, soil organic matter; pH, soil pH.

The relationship between AM fungal communities and soil properties was analyzed at the genus level (Figure 6). A redundancy analysis (RDA) was conducted to assess the influences of soil factors on the AM fungal community across all soil samples. The first two ordinal axes of the RDA accounted for 72.02 and 20.42% of the total variation in the soil AM fungal community from 2020, respectively (Figure 6A). The first axis was negatively correlated with soil TN, AK, SOM, and pH, while showing a positive correlation with soil TP. The second axis exhibited a positive correlation with soil TN, TP, and SOM, and a negative correlation with soil AK and pH. The most significant soil factors were pH (R^2^ = 0.77, *p* < 0.001) and TN (R^2^ = 0.48, *p* < 0.001) (Figure 6A; Supplementary Table S3).

We also employed Spearman’s rank correlation to evaluate the relationships among the different AM fungal genera and soil properties in 2020 (Figure 6B; Supplementary Table S4). *Acaulospora* was significantly negatively correlated with pH (*R*^2^ = −0.36, *p* = 0.012). *Ambispora* was significantly negatively correlated with SOM (*R*^2^ = −0.35, *p* = 0.014), AK (*R*^2^ = −0.38, *p* = 0.008), and pH (*R*^2^ = −0.46, *p* = 0.001). *Claroideoglomus*, *Diversispora*, and *Entrophospora* were significantly positively correlated with TN, SOM, and pH (*p* < 0.05). In contrast, *Glomus* and *Paralomus* were significantly negatively correlated with TN, SOM, and pH (*p* < 0.05). Additionally, *Claroideoglomus* and *Entrophospora* were significantly positively correlated with AK, while *Paralomus* was significantly negatively correlated (*p* < 0.05). Others were significantly positively correlated with soil TN (*R*^2^ = 0.34, *p* = 0.020), SOM (*R*^2^ = 0.39, *p* = 0.007), and pH (*R*^2^ = 0.55, *p* < 0.001), while negatively correlated with TP (*R*^2^ = −0.33, *p* = 0.023).

The first two ordinal axes of RDA accounted for 59.80 and 34.14% of the total variation in the soil AM fungal community from 2021, respectively (Figure 6C). The first axis was positively correlated with soil TP and AK, but negatively correlated with soil TN, SOM, and pH. The second axis was positively correlated with soil TP, AK, SOM, and TN, while negatively correlated with soil pH. The most significant soil factors were TN (*R*^2^ = 0.79, *p* < 0.001), pH (*R*^2^ = 0.77, *p* < 0.001), and SOM (*R*^2^ = 0.73, *p* < 0.001) (Figure 6C; Supplementary Table S3). Spearman’s rank correlation was employed to assess the relationship among the abundant AM fungal genera and soil properties in 2021 (Figure 6D; Supplementary Table S4). Overall, the correlation between AM fungi (*Acaulospora*, *Ambispora*, *Claroideoglomus*, *Diversispora*, and *Entrophospora*) and TN, TP, SOM, and pH in 2021 was similar to that in 2020, but with stronger correlations observed in 2021. *Glomus* was significantly negatively correlated with TP (*p* < 0.05), and *Paraglomus* was positively correlated with SOM and AK, which was exactly the opposite of the results in 2020.

**Figure 7:**
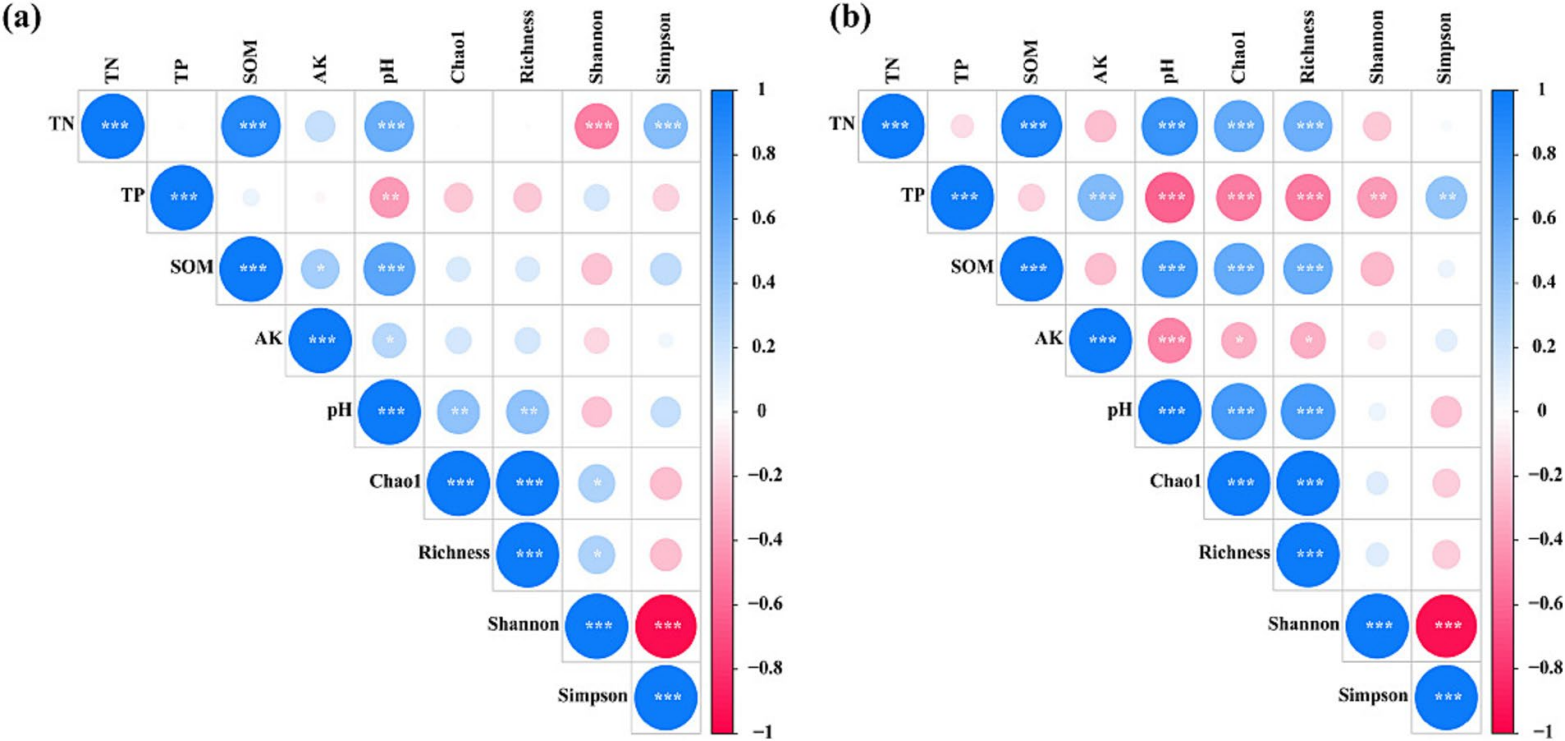
Correlation coefficient matrix. Factors influencing the AM fungal community were analyzed using Pearson correlation (*p* < 0.05 *, *p* < 0.01 **, *p* < 0.001 ***). Red colors indicate negative correlations blue colors indicate positive connections. Darker color represents a greater correlation coefficient. **(A)** 2020, **(B)** 2021. TN, total nitrogen; TP, total phosphorus; AK, available potassium; SOM, soil organic matter; pH, soil pH.

Furthermore, we developed a correlation coefficient matrix to examine the relationship between soil factors and the diversity and richness of AM fungal communities (Figure 7). Soil TN was significantly positively correlated with the Simpson index of the AM fungal community, whereas it was significantly negatively correlated with the Shannon index in 2020 (*p* < 0.05). Soil TN, TP, SOM, and pH were significantly positively correlated with the Chao1 and Richness indices, while TP and AK were significantly negatively correlated with the Chao1 and Richness indices (*p* < 0.05). Additionally, soil TP was significantly positively correlated with the Simpson index, while it was significantly negatively correlated with the Shannon index (*p* < 0.05).

## 4 Discussion

Extreme habitats often host groups of organisms with unique physiological and ecological functions. The Tibetan plateau alpine grassland ecosystem is a typical extreme habitat, making the study of AM fungal diversity particularly significant. In this research, we analyzed the characteristics and seasonal dynamics of soil AM fungal communities in Hongyuan and Ruoergai. We determined the diversity of AM fungal communities and their response mechanisms to seasonal variation in different degraded grasslands on the eastern Tibetan Plateau. The findings suggest that both spatial (location-specific) and temporal (interannual and seasonal) factors significantly influence the diversity and composition of AM fungal communities.

### 4.1 The AM fungal species

A total of 97 species of AM fungi were identified in this study, representing approximately 66.90% of the known species of AM fungi in China, indicating that the soil of Hongyuan and Ruoergai are abundant in AM fungal resources. The 97 AM fungi identified belong to 10 genera: *Acaulospora*, *Archaeospora*, *Claroideoglomus*, *Diversispora*, *Entrophospora*, *Gigaspora*, *Glomus*, *Pacispora*, *Paraglomus*, and *Scutellospora*, all of which have been previously reported in the Tibetan Plateau (Gai et al., 2009; Mao et al., 2024; Zhang et al., 2024). Among these, *Glomus* and *Paraglomus* were the dominant genera, detected in all soil samples, with relative abundances of 15.73 and 15.60%, respectively. The spatial distribution of AM fungal communities in 15 alpine grasslands on the Tibetan Plateau showed that *Glomus* was the dominant genus in alpine meadow and alpine steppe, followed by *Paraglomus* (Zhang et al., 2024). *Glomus* has repeatedly been shown to be widely distributed in different ecosystems worldwide and to be highly adaptable to extreme environments (Coutinho et al., 2015; Bonfim et al., 2016). Additionally, *Acaulospora* occurred at different sites and seasons, although its relative abundance was below 1% in this study. It has been found to have a higher occurrence frequency in alpine grasslands (Gai et al., 2009), and its relative abundance gradually increases with elevation (3105–4,556 m) (Li et al., 2018). This result further confirms that *Acaulospora* is more suited to survive in high-altitude alpine grasslands.

We also found that the AM fungal taxa exhibiting significant changes mainly belonged to Paraglomeraceae (*Paraglomus*), Gigasporaceae (*Scutellospora*), Glomeraceae (*Glomus*), Acaulosporaceae (*Acaulospora*), and Ambisporaceae (*Ambispora*) in Hongyuan, as well as Diversisporaceae (*Diversispora*), Claroideoglomeraceae (*Claroideoglomus*), Pacisporaceae (*Pacispora*), Glomeraceae (*Glomus*), *Entrophospora*, and Archaeosporaceae (*Archaeospora*) in Ruoergai (Figures 4A,B). This indicates that these AM fungal taxa are highly sensitive to changes in the external environment. Future research should focus on these AM fungal taxa, in particularly *Glomus* and *Paraglomus*.

### 4.2 Changes in AM fungal community characteristics

Seasonal variation is an important factor influencing AM fungal communities, but its role in the alpine grassland ecosystem on the Tibetan Plateau is still lacking. In this study, we found seasonal variation affected the species, community structure, composition, diversity, and network complexity of AM fungi in Hongyuan and Ruoergai. The results are consistent with previous findings in other grassland ecosystems. For example, Dumbrell et al. (2011) found that the composition and structure of AM fungal communities in temperate grassland significantly changed with seasonal variations. Su et al. (2011) explored the seasonal variation of AM fungi in the rhizosphere of five plant species in Inner Mongolia grassland, and found that seasons had significant effects on the spore density, Richness, and Shannon index of AM fungi. Our hypothesis that seasonal variations are a crucial factor affecting the AM fungal community in the alpine grasslands of the eastern Tibetan Plateau, and this viewpoint is supported. Previous studies have shown that the response of AM fungal communities to seasonal variation differs and is affected by plant species and habitat (Helgason et al., 2014). In this study, we found that the response of AM fungal communities to seasonal variation is also affected by site-specific environmental factors, as evidenced by the contrasting results of Shannon index and the network complexity of AM fungal communities in Hongyuan soils and Ruoergai soils in 2021. Therefore, we speculate that, in addition to seasonal variations, geographical distance is also an important factor influencing the AM fungal communities in the eastern Tibetan Plateau. Previous studies have indicated that geographic distance significantly affects the community dynamics of AM fungi in the Tibetan Plateau (Zhang et al., 2024), and has a more significant impact on fungal communities compared to other environmental factors (Kivlin et al., 2011; Beck et al., 2015). This phenomenon is also known as the distance decay rule (Wang et al., 2022).

However, some studies have found the community composition, richness, and spore density of AM fungi do not change significantly with seasonal variations (Pereira et al., 2018; Álvarez-Lopeztello et al., 2019). Thus, the effect of seasonal variation on the characteristics of AM fungal communities is complex and influenced by a various of environmental factors. Therefore, further research is necessary to clarify the impact of climate and environmental factors on the characterization of AM fungal communities in these ecosystems.

### 4.3 Correlations of changes in AM fungal community with soil properties

Soil, as an important medium for the interaction between plants and AM fungi, is usually regarded as a key environmental factor (Davison et al., 2015; Wang et al., 2021). In this study, seasonal variations significantly impacted the TN, TP, SOM, and pH of the soils in Hongyuan and Ruoergai. The changes in these indicators were significantly correlated with the variations in AM fungal communities, and similar patterns of change were observed across different years. A comprehensive analysis of the results indicated that soil TN, SOM, and pH were the key soil factors influencing the characteristics of AM fungal communities. In previous studies, TN and pH have also been identified as crucial soil factors influencing the structure of AM fungal communities (Guo and Gong, 2014; Ma et al., 2021). Additionally, the pH can affect the root colonization of AM fungi and determine their niche space (Van Aarle et al., 2002; Davison et al., 2021). The compounds produced by the decomposition of SOM contribute to the colonization and hyphal growth of AM fungi (Gryndler et al., 2009). Moreover, AM fungi can, in turn, inhibit the decomposition of SOM by competing with saprophytic fungi (Frey, 2019), or increase soil TN content by reducing nitrogen leaching (Fang et al., 2020). Therefore, we propose that the response of soil AM fungal community characteristics to seasonal variation in the eastern Tibetan Plateau is linked to soil properties.

## 5 Conclusion

Through high-throughput sequencing, the present study reveals the diversity, seasonal dynamics, and soil driving factors of AM fungal communities in the eastern Tibetan Plateau. A total of 937 OTUs were detected, belonging to 4 orders, 9 families, 10 genera, and 97 species, suggesting that the research sites possess a high abundance and rich diversity of AM fungi. Seasonal variation exerts a significantly impact on the community structure, composition, diversity, and network complexity of AM fungi. Moreover, the influence of this seasonal variation is modulated by geographic distance and year. Additionally, soil properties also play a vital role as factors influencing AM fungal communities. Among these properties, total nitrogen, soil organic matter and pH are the key factors. The dominant AM fungi genera, such as *Glomus* and *Paraglomus*, are highly sensitive to seasonal variation and thus deserve particular attention in ecological monitoring as well as grassland restoration efforts.

## Data Availability

The datasets presented in this study can be found in online repositories. The names of the repository/repositories and accession number(s) can be found at: https://www.ncbi.nlm.nih.gov/, PRJNA1173197.

## Footnotes

^1^ https://www.bioincloud.tech
