# Supplementary material for: Diversity of arbuscular mycorrhizal fungi and its response to seasonal variation in alpine grassland of the eastern Tibetan Plateau
Source: Front Microbiol. 2025 Feb 25;16:1511979. doi: 10.3389/fmicb.2025.1511979 (PMC11893506; doi:10.3389/fmicb.2025.1511979)
Supplement: Supplementary file 1 [file Data_Sheet_1.docx]

**Diversity of arbuscular mycorrhizal fungi and its response to seasonal variation in alpine grassland of the eastern Tibetan Plateau**

Wanqing Dong^1, 2, 3^, Tingting Ding^1, 2, 3^ and Tingyu Duan^1, 2, 3*^

1. State Key Laboratory of Herbage Improvement and Grassland Agro-ecosystems, Lanzhou University, Lanzhou, China

2. Key Laboratory of Grassland Livestock Industry Innovation, Ministry of Agriculture and Rural Affairs; Engineering Research Center of Grassland Industry, Ministry of Education; Gansu Tech Innovation Center of Western China Grassland Industry, Lanzhou, China

3. College of Pastoral Agriculture Science and Technology, Lanzhou University, Lanzhou, China

*** Correspondence:**Tingyu Duan
[duanty@lzu.edu.cn](mailto:duanty@lzu.edu.cn)

**Supplementary material**

**Figure S1** Rarefaction curves at the OTU level (Based on Richness)

**
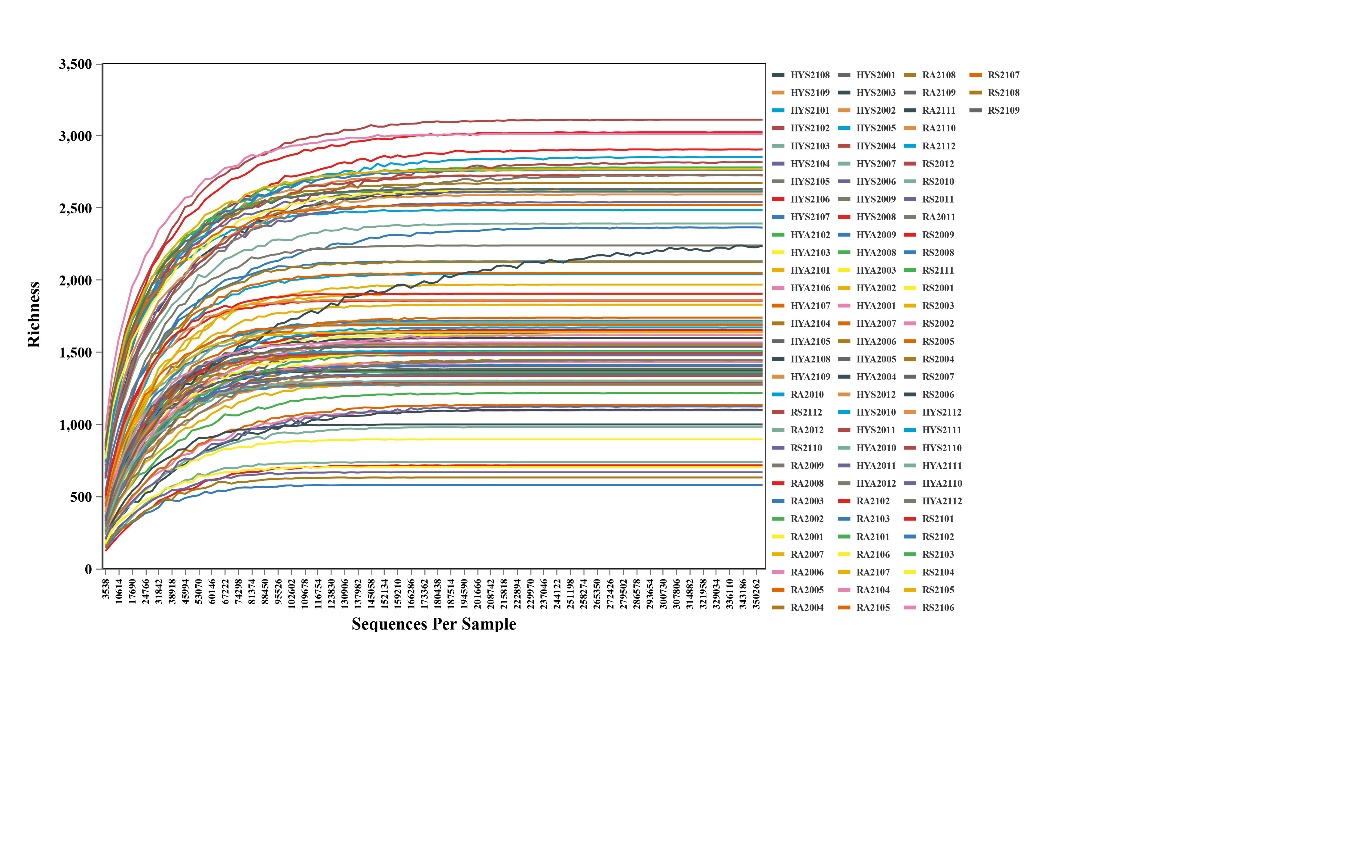
**

**Figure S2** Distribution of AM fungi in different genera

**
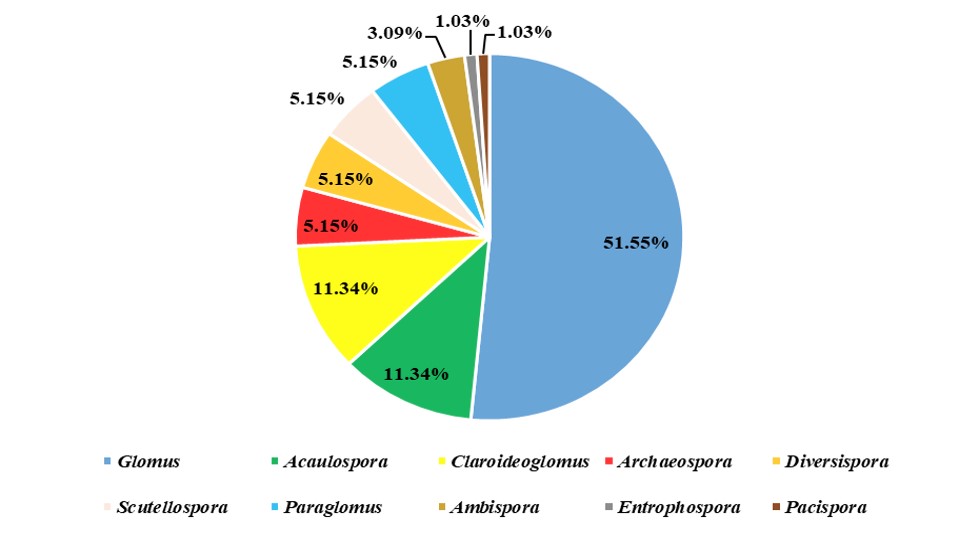
**

**Figure S3** The number of AM fungal species in different groups of soil samples. Significant differences among treatments for each variable were assessed using Tukey’s honestly significant difference (HSD) test (*P*<0.05) following one-way ANOVA and are indicated by different letters. HYS20, HYA20, RS20, RA20, HYS21, HYA21, RS21 and RA21 represent different groups of soil samples.

**
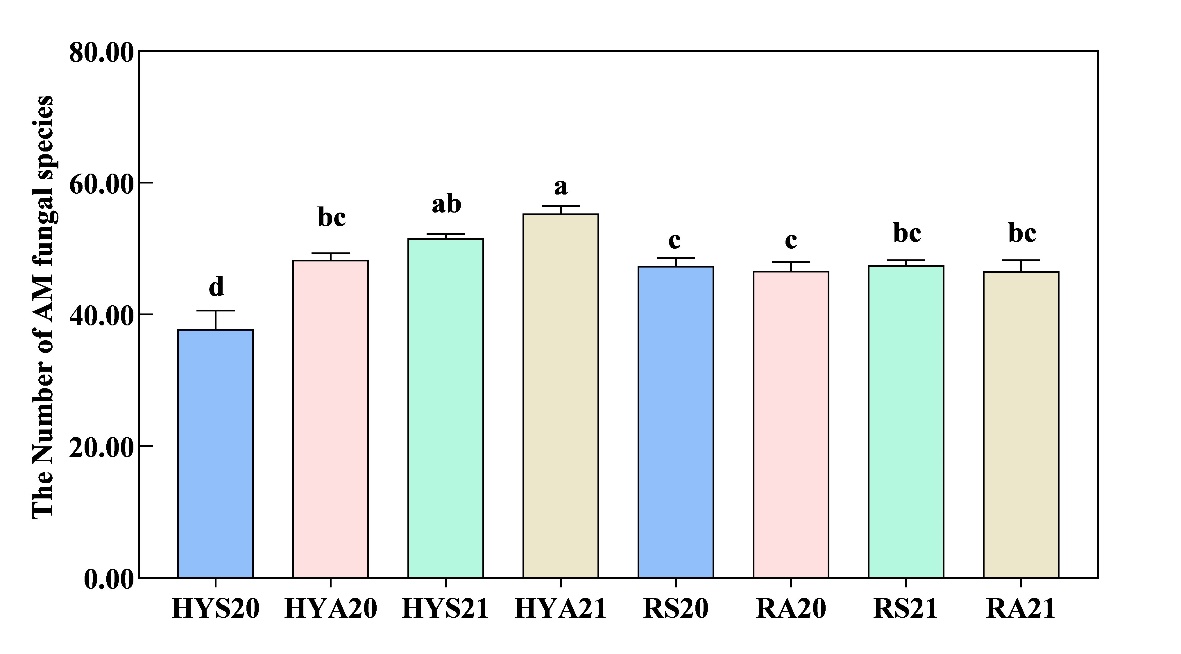
**

**Figure S4** The Cladogra of LEfSe analysis. HYS20, HYA20, RS20, RA20, HYS21, HYA21, RS21 and RA21 represent different groups of soil samples.

**
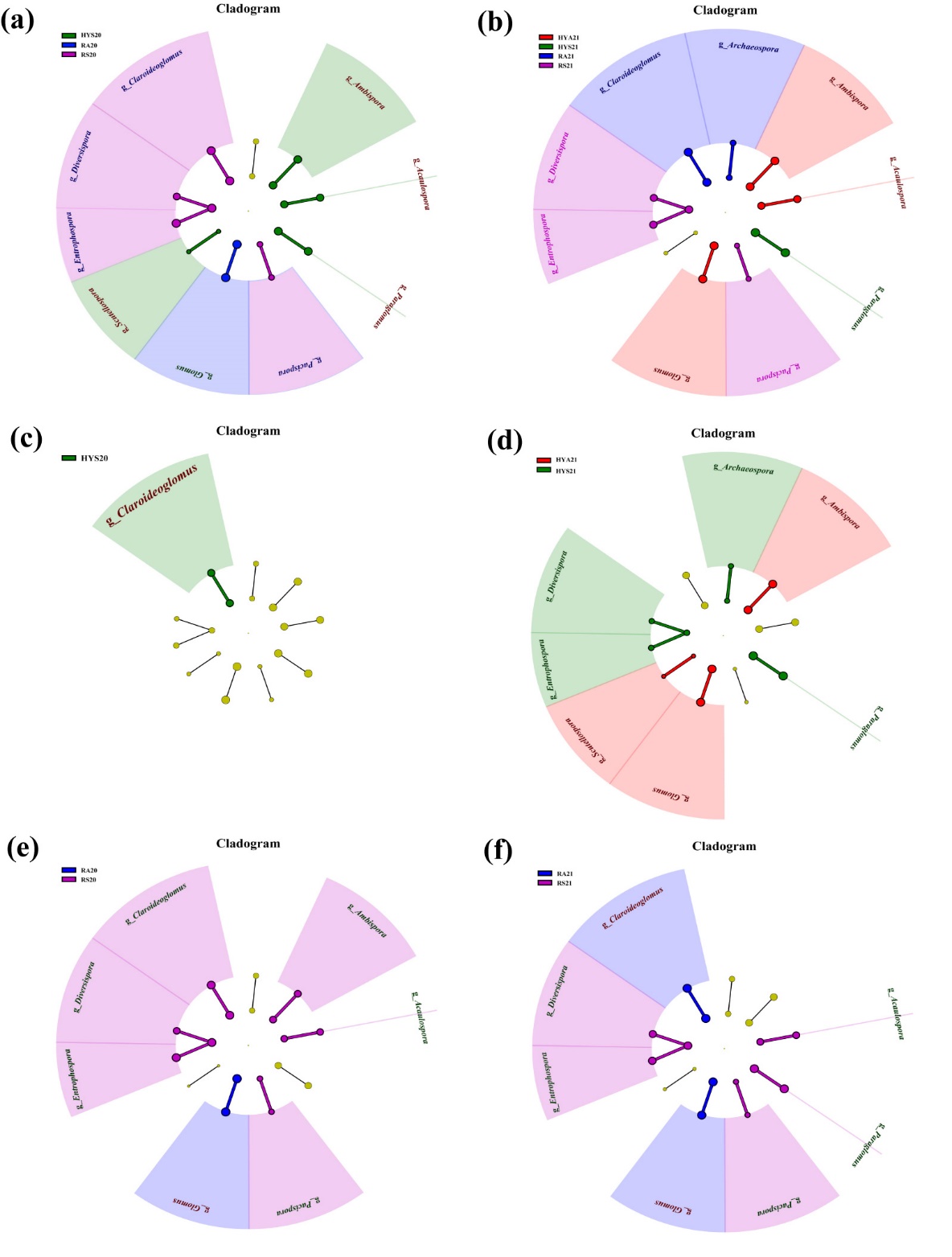
**

**Table S1** Relative abundance of AM fungal communities at the order, family, and genus level in all samples. Taxa with abundance <1% are summarized as “others”.

| Taxonomy | Relative abundance (%) |
| --- | --- |
| **Order** |  |
| Paraglomerales | 24.42 |
| Glomerales | 23.36 |
| Diversisporales | 6.18 |
| Archaeosporales | 2.81 |
| Unclassified | 43.23 |
| **Family** |  |
| Paraglomeraceae | 17.28 |
| Glomeraceae | 15.79 |
| Claroideoglomeraceae | 5.10 |
| Diversisporaceae | 4.64 |
| Ambisporaceae | 2.71 |
| Others | 1.53 |
| Unclassified | 52.95 |
| **Genus** |  |
| *Glomus* | 15.73 |
| *Paraglomus* | 15.60 |
| *Claroideoglomus* | 5.06 |
| *Diversispora* | 2.49 |
| *Entrophospora* | 2.15 |
| *Ambispora* | 2.71 |
| Others | 1.52 |
| Unclassified | 54.74 |

**Table S2** The distribution of AM fungi at the order, family, genus, and species level among different sites and seasons in soil samples from 2020 and 2021. HYS20, HYA20, RS20, RA20, HYS21, HYA21, RS21 and RA21 represent different groups of soil samples.

| Order | Family | Genus | Species | 2020 | | | | 2021 | | | |
| --- | --- | --- | --- | --- | --- | --- | --- | --- | --- | --- | --- |
|  |  |  |  | HYS20 | RS20 | HYA20 | RA20 | HYS21 | RS21 | HYA21 | RA21 |
| Archaeosporales | Ambisporaceae | *Ambispora* | *Ambispora fennica* | * | * | * | * | * | * | * | * |
|  |  |  | *Ambispora leptoticha* |  |  | * | * |  |  |  |  |
|  |  |  | *Ambispora* Liu2012a_Ar_1 |  |  |  |  | * | * |  |  |
|  | Archaeosporaceae | *Archaeospora* | *Archaeospora* Aca | * | * | * | * | * | * | * | * |
|  |  |  | *Archaeospora* MO_Ar1 | * | * |  | * |  |  |  | * |
|  |  |  | *Archaeospora* Other1 | * |  |  |  | * | * | * |  |
|  |  |  | *Archaeospora* Schechter08_Arch1 |  | * |  | * | * | * |  |  |
|  |  |  | *Archaeospora* sp | * | * |  |  | * | * |  |  |
| Diversisporales | Acaulosporaceae | *Acaulospora* | *Acaulospora* Acau10 | * | * |  |  |  |  |  |  |
|  |  |  | *Acaulospora* Acau2 | * | * | * | * | * | * | * | * |
|  |  |  | *Acaulospora* Acau3 | * | * | * | * | * | * | * | * |
|  |  |  | *Acaulospora* Acau4 | * |  |  |  | * |  |  |  |
|  |  |  | *Acaulospora* Acau8 |  |  |  |  | * | * |  |  |
|  |  |  | *Acaulospora* MO_A10 | * | * | * |  | * | * | * | * |
|  |  |  | *Acaulospora* MO_A3 | * | * | * | * | * | * | * | * |
|  |  |  | *Acaulospora* MO_A4 | * | * | * | * | * | * | * | * |
|  |  |  | *Acaulospora* MO_A5 | * | * |  |  | * | * | * | * |
|  |  |  | *Acaulospora* MO_A8 | * | * |  |  |  |  | * | * |
|  |  |  | *Acaulospora* sp | * | * | * | * | * | * | * | * |
|  | Diversisporaceae | *Diversispora* | *Diversispora* Clade_3 | * | * | * | * | * | * |  |  |
|  |  |  | *Diversispora* Div |  | * |  | * |  |  |  |  |
|  |  |  | *Diversispora* MO_GC1 |  | * | * | * |  |  |  | * |
|  |  |  | *Diversispora* sp | * | * | * | * | * | * | * | * |
|  |  |  | *Diversispora trimurales* | * | * | * | * | * | * | * | * |
|  |  | *Entrophospora* | *Entrophospora baltica* | * | * | * | * | * | * | * | * |
|  | Gigasporaceae | *Scutellospora* | *Scutellospora* MO_S2 | * | * |  |  |  |  |  |  |
|  |  |  | *Scutellospora nodosa* |  |  |  |  |  |  | * | * |
|  |  |  | *Scutellospora projecturata* |  |  | * |  |  |  |  |  |
|  |  |  | *Scutellospora* Scut1 | * |  | * |  |  |  | * | * |
|  |  |  | *Scutellospora spinosissima* |  |  |  |  |  |  |  | * |
|  | Pacisporaceae | *Pacispora* | *Pacispora scintillans* | * | * | * | * | * | * |  | * |
| Glomerales | Claroideoglomeraceae | *Claroideoglomus* | *Claroideoglomus* acnaGlo7 | * | * | * | * | * | * | * | * |
|  |  |  | *Claroideoglomus* Alguacil12b_GLO_G3 | * | * | * | * | * | * | * | * |
|  |  |  | *Claroideoglomus* Douhan9 | * | * | * | * | * | * | * | * |
|  |  |  | *Claroideoglomus* GlBb12 | * | * | * | * | * | * | * | * |
|  |  |  | *Claroideoglomus* Glo_G8 | * | * | * | * | * | * | * | * |
|  |  |  | *Claroideoglomus* Glo58 | * | * | * | * | * | * | * | * |
|  |  |  | *Claroideoglomus* Glo59 | * | * | * | * | * | * | * | * |
|  |  |  | *Claroideoglomus lamellosum* | * | * | * | * | * | * | * | * |
|  |  |  | *Claroideoglomus* MO_GB1 | * | * | * | * |  |  | * | * |
|  |  |  | *Claroideoglomus* ORVIN_GLO4 |  |  |  | * |  |  |  |  |
|  |  |  | *Claroideoglomus* Torrecillas12b_Glo_G5 |  | * | * | * | * | * | * | * |
|  | Glomeraceae | *Glomus* | *Glomus* Alguacil09b_Glo_G8 | * | * | * | * |  |  | * | * |
|  |  |  | *Glomus* Alguacil11d_Glo_G8 |  |  |  |  | * | * |  |  |
|  |  |  | *Glomus caledonium* | * | * | * | * | * | * | * | * |
|  |  |  | *Glomus* Douhan3 | * | * | * | * | * | * | * | * |
|  |  |  | *Glomus* Franke_A1 |  |  | * | * |  |  |  |  |
|  |  |  | *Glomus* GlAb42 |  |  | * | * |  |  |  |  |
|  |  |  | *Glomus* GlAd22 |  |  |  |  | * | * |  |  |
|  |  |  | *Glomus* Glo_A | * | * | * | * | * | * | * | * |
|  |  |  | *Glomus* Glo_E | * |  | * | * | * | * | * | * |
|  |  |  | *Glomus* Glo_I |  |  |  |  |  |  |  | * |
|  |  |  | *Glomus* Glo3 | * | * | * | * | * | * | * | * |
|  |  |  | *Glomus* Glo49 |  |  | * | * |  |  |  |  |
|  |  |  | *Glomus* Glo7 | * | * | * | * | * | * | * | * |
|  |  |  | *Glomus* Liu2012b_Phylo_17 | * | * | * | * | * | * | * | * |
|  |  |  | *Glomus* Liu2012b_Phylo_5 | * | * | * | * | * | * | * | * |
|  |  |  | *Glomus* MO_G1 | * | * | * | * | * | * | * | * |
|  |  |  | *Glomus* MO_G14 | * | * | * | * | * | * | * | * |
|  |  |  | *Glomus* MO_G15 | * | * | * | * | * | * | * | * |
|  |  |  | *Glomus* MO_G16 |  |  |  |  | * | * |  |  |
|  |  |  | *Glomus* MO_G17 |  |  |  |  |  |  | * | * |
|  |  |  | *Glomus* MO_G18 | * | * | * | * | * | * | * | * |
|  |  |  | *Glomus* MO_G20 | * | * | * | * | * | * | * | * |
|  |  |  | *Glomus* MO_G21 | * | * | * | * | * | * | * | * |
|  |  |  | *Glomus* MO_G22 | * | * | * | * | * | * | * | * |
|  |  |  | *Glomus* MO_G23 | * | * | * | * | * | * | * | * |
|  |  |  | *Glomus* MO_G27 |  |  | * | * | * | * | * | * |
|  |  |  | *Glomus* MO_G31 | * | * | * | * | * | * | * | * |
|  |  |  | *Glomus* MO_G4 | * | * | * | * |  |  | * | * |
|  |  |  | *Glomus* MO_G50 |  |  | * | * |  |  |  |  |
|  |  |  | *Glomus* MO_G51 |  |  | * |  |  |  |  |  |
|  |  |  | *Glomus* MO_G56 |  |  | * | * |  |  |  |  |
|  |  |  | *Glomus* MO_G60 |  |  | * | * |  |  |  |  |
|  |  |  | *Glomus* MO_G7 | * | * | * | * | * | * | * | * |
|  |  |  | *Glomus* NES27 | * |  | * |  |  |  | * | * |
|  |  |  | *Glomus* NF13 | * | * | * | * | * | * | * | * |
|  |  |  | *Glomus* ORVIN_GLO1E |  |  | * | * |  |  |  |  |
|  |  |  | *Glomus* ORVIN_GLO3B |  |  | * | * |  |  |  |  |
|  |  |  | *Glomus* ORVIN_GLO3D |  |  |  |  | * | * |  |  |
|  |  |  | *Glomus* ORVIN_GLO3E | * | * |  | * |  |  |  |  |
|  |  |  | *Glomus* PF19 | * | * | * | * |  |  |  |  |
|  |  |  | *Glomus* PF22 | * | * | * | * | * | * | * | * |
|  |  |  | *Glomus* PSAMG1 |  |  | * | * |  |  |  |  |
|  |  |  | *Glomus* PSAMG2 |  |  | * |  |  |  |  |  |
|  |  |  | *Glomus* sp | * | * | * | * | * | * | * | * |
|  |  |  | *Glomus* Whitfield_type_17 |  | * |  | * |  |  |  | * |
|  |  |  | *Glomus* Wirsel_OTU12 | * | * | * | * | * | * | * | * |
|  |  |  | *Glomus* Wirsel_OTU14 | * | * | * | * | * | * | * | * |
|  |  |  | *Glomus* Wirsel_OTU6 |  |  |  |  | * | * |  |  |
|  |  |  | *Glomus* Yamato2005_D |  |  | * | * |  |  |  |  |
|  |  |  | *Glomus* Yamato2005_E | * | * | * | * | * | * | * | * |
| Paraglomerales | Paraglomeraceae | *Paraglomus* | *Paraglomus* Alguacil12a_Para_1 | * | * | * | * | * | * | * | * |
|  |  |  | *Paraglomus* Alguacil12b_ACA1 | * | * | * | * | * | * | * | * |
|  |  |  | *Paraglomus* *brasilianum* | * |  |  |  |  |  | * |  |
|  |  |  | *Paraglomus* Glom_1B13 | * |  | * | * |  |  | * | * |
|  |  |  | *Paraglomus* sp | * | * | * | * |  |  | * | * |

**Table S3** Redundancy analysis (RDA) at the genus level, utilized to examine the correlation between AM fungal communities and soil properties in 2020 and 2021. TN, total nitrogen; TP, total phosphorus; AK, available potassium; SOM, soil organic matter; pH, soil pH.

| Year | Soil factors | RDA1 | RDA2 | R^2^ | *p*-value |
| --- | --- | --- | --- | --- | --- |
| 2020 | TN | -0.916 | 0.400 | 0.483 | **<0.001** |
|  | TP | 0.993 | 0.115 | 0.102 | 0.085 |
|  | SOM | -0.993 | 0.118 | 0.326 | **0.001** |
|  | AK | -0.873 | -0.488 | 0.126 | **0.049** |
|  | pH | -0.994 | -0.107 | 0.772 | **<0.001** |
| 2021 | TN | -1.000 | 0.007 | 0.791 | **<0.001** |
|  | TP | 0.517 | 0.856 | 0.359 | **<0.001** |
|  | SOM | -0.995 | 0.099 | 0.731 | **<0.001** |
|  | AK | 0.773 | 0.634 | 0.113 | 0.057 |
|  | pH | -0.955 | -0.296 | 0.768 | **<0.001** |

**Table S4** Spearman's rank correlation heatmap, employed to study the correlation between AM fungal communities and soil properties in 2020 and 2021. Taxa with abundance <1% are summarized as “others”. TN, total nitrogen; TP, total phosphorus; AK, available potassium; SOM, soil organic matter; pH, soil pH.

| Year | Soil factors | *Acaulospora* | | *Ambispora* | | *Claroideoglomus* | | *Diversispora* | | *Entrophospora* | | *Glomus* | | *Paraglomus* | | others | |
| --- | --- | --- | --- | --- | --- | --- | --- | --- | --- | --- | --- | --- | --- | --- | --- | --- | --- |
|  |  | R^2^ | *p*-value | R^2^ | *p*-value | R^2^ | *p*-value | R^2^ | *p*-value | R^2^ | *p*-value | R^2^ | *p*-value | R^2^ | *p*-value | R^2^ | *p*-value |
| 2020 | TN | -0.065 | 0.661 | -0.195 | 0.185 | 0.611 | **<0.001** | 0.455 | **0.001** | 0.500 | **<0.001** | -0.541 | **<0.001** | -0.443 | **0.002** | 0.336 | **0.020** |
|  | TP | 0.239 | 0.102 | 0.217 | 0.138 | -0.261 | 0.073 | -0.227 | 0.120 | -0.109 | 0.460 | 0.228 | 0.119 | 0.218 | 0.136 | -0.328 | **0.023** |
|  | SOM | -0.256 | 0.079 | -0.352 | **0.014** | 0.685 | **<0.001** | 0.555 | **<0.001** | 0.501 | **<0.001** | -0.405 | **0.004** | -0.488 | **<0.001** | 0.386 | **0.007** |
|  | AK | -0.195 | 0.185 | -0.381 | **0.008** | 0.363 | **0.011** | 0.279 | 0.055 | 0.355 | **0.013** | -0.186 | 0.205 | -0.330 | **0.022** | 0.267 | 0.066 |
|  | pH | -0.358 | **0.012** | -0.459 | **0.001** | 0.791 | **<0.001** | 0.782 | **<0.001** | 0.705 | **<0.001** | -0.410 | **0.004** | -0.687 | **<0.001** | 0.553 | **<0.001** |
| 2021 | TN | -0.536 | **<0.001** | -0.708 | **<0.001** | 0.667 | **<0.001** | 0.675 | **<0.001** | 0.462 | **0.001** | -0.105 | 0.477 | -0.074 | 0.618 | 0.325 | **0.024** |
|  | TP | 0.168 | 0.253 | 0.092 | 0.536 | -0.339 | **0.018** | -0.135 | 0.361 | 0.084 | 0.571 | -0.452 | **0.001** | 0.454 | **0.001** | -0.038 | 0.800 |
|  | SOM | -0.510 | **<0.001** | -0.724 | **<0.001** | 0.596 | **<0.001** | 0.666 | **<0.001** | 0.542 | **<0.001** | -0.220 | 0.133 | 0.069 | 0.642 | 0.421 | **0.003** |
|  | AK | -0.010 | 0.947 | 0.144 | 0.329 | -0.226 | 0.123 | -0.177 | 0.229 | -0.198 | 0.176 | -0.164 | 0.264 | 0.219 | 0.134 | 0.026 | 0.863 |
|  | pH | -0.291 | **0.045** | -0.561 | **<0.001** | 0.678 | **<0.001** | 0.624 | **<0.001** | 0.347 | **0.016** | 0.185 | 0.208 | -0.326 | **0.024** | 0.197 | 0.180 |
